# Modeling the Habitat Suitability and Range Shift of *Daphniphyllum macropodum* in China Under Climate Change Using an Optimized MaxEnt Model

**DOI:** 10.3390/biology14101360

**Published:** 2025-10-03

**Authors:** Yangzhou Xiang, Suhang Li, Qiong Yang, Jiaojiao Liu, Ying Liu, Ling Zhao, Hua Lin, Yang Luo, Jun Ren, Xuqiang Luo, Hua Wang

**Affiliations:** 1 School of Geography and Resources, Guizhou Education University, Guiyang 550018, China; 2 School of Biological Sciences, Guizhou Education University, Guiyang 550018, China; 3 State Power Investment Corporation Power Station Operation Technology (Beijing) Co., Ltd., Beijing 100032, China; 4 Guizhou Institute of Forest Inventory and Planning, Guiyang 550003, China

**Keywords:** *Daphniphyllum macropodum*, climate change, MaxEnt optimization model, potential geographic distribution, centroid shift

## Abstract

Climate change continues to threaten global biodiversity, making it essential to assess how keystone species may shift their distributions and to use these findings to inform conservation planning. This study evaluated the current and future habitat suitability of *D. macropodum*, an important tree species within subtropical evergreen broad-leaved forests in China, using 354 occurrence records and a suite of environmental variables. A parameter-optimized MaxEnt model (calibrated with ENMeval; RM = 4, FC = QHPT) was applied to simulate the species’ present distribution and projected changes under three climate scenarios (SSP126, SSP245, SSP585). The main factors influencing distribution were determined to be moisture and temperature seasonality, with the precipitation of the coldest quarter (Bio19, 36.3%), the mean diurnal range (Bio2, 37.5%), and the precipitation of the warmest quarter (Bio18, 14.2%) jointly contributing 88.0% of the total influence. The model projections indicated a 40.1% reduction in the total number of suitable habitats under high-emission scenarios (SSP585) by the 2090s, including a loss of over 80% of highly suitable areas. Centroid movements also diverged across the scenarios: a southwestern shift under SSP126 and SSP245 contrasted with a southeastern shift under SSP585, with each accompanied by significant habitat fragmentation. Key climate refugia were identified primarily in central Taiwan Province and the mountainous zones of Zhejiang and Fujian Provinces, which should be prioritized for conservation activities. These insights offer a foundational understanding for the conservation of *D. macropodum* and other ecologically similar subtropical evergreen species. However, direct extrapolation to other taxa should be made cautiously, as specific responses may vary based on differing ecological tolerances and dispersal capacities. Further research is needed to test the generalizability of these patterns across diverse plant functional types.

## Simple Summary

Climate change threatens plant species globally, necessitating predictions of habitat shifts. This study examines *Daphniphyllum macropodum*, a key tree in China’s subtropical evergreen forests. Using an optimized model integrating species occurrences, climate, terrain, and human impacts, we mapped this species’ current habitat and projected its future distributions. The results indicate that seasonal precipitation and temperature are its primary distribution drivers. Under high emissions, suitable habitats may decline by over 40% by 2100, with high-suitability areas decreasing by more than 80%. Habitat fragmentation is projected to intensify, and the core range may shift southwest or southeast. Key refugia include central Taiwan and the mountains of Zhejiang and Fujian, which merit urgent conservation efforts. These insights aid biodiversity protection and assisted-migration planning, supporting practical forest management.

## 1. Introduction

Global climate change, characterized by rising atmospheric CO_2_ concentrations, increasing temperatures, and altered precipitation patterns [1], is recognized as one of the most severe threats to biodiversity in the 21st century [2,3]. These impacts are particularly acute in biodiverse yet vulnerable regions such as the subtropical forests of China, which are experiencing rapid climatic shifts [4]. These anthropogenically driven shifts in the climate system are profoundly affecting species survival and distributions at an unprecedented rate and magnitude [5,6]. As fundamental units of ecosystems, shifts, contractions, and fragmentation of species’ geographic ranges are considered direct responses to climatic stressors, and they may trigger further cascading effects, including the disruption of community structures, breakdown of ecological interactions, and degradation of ecosystem services [7]. Therefore, developing high-accuracy models to simulate the dynamic responses of species distributions to future climate scenarios has become a central focus in macroecology and conservation biology, providing critical decision support for the formulation of proactive biodiversity conservation strategies. Species with narrow climatic niches and high sensitivity to environmental change, such as *Daphniphyllum macropodum*, serve as critical bioindicators. Their distributional responses provide early and clear signals of ecosystem-level changes [8], making them ideal models for projecting climatic impacts on subtropical Asian forests.

China is regarded as a critical region for global biodiversity conservation [9], and its complex ecosystems are considered highly vulnerable to climate change [10,11]. Against this background, systematic assessment of the climatic response mechanisms of keystone species has been identified as a priority in national strategies for ecological security and sustainable resource management [12,13,14]. *Daphniphyllum macropodum* Miq., a phylogenetically ancient tree species in the family *Daphniphyllaceae*, is often found as a dominant companion or foundational species in subtropical evergreen broad-leaved forests [15,16], playing a significant role in maintaining community structure and functional stability [17]. This species is noted not only for its broad ecological adaptability but also for its strong capacity for sprout regeneration, which enables it to facilitate natural recovery in degraded ecosystems [18]. Furthermore, its tissues are known to contain abundant alkaloids and endophytic fungi with broad-spectrum antimicrobial activity, indicating their considerable potential for pharmaceutical and biopesticide applications [19]. The species is also valued for its ornamental and cultural functions, owing to its straight trunk, elegant canopy, and purplish-red petioles and veins [20]. It is primarily distributed in humid valley slopes at elevations of 600–1700 m [21] and is highly sensitive to changes in hydrothermal conditions [22], making it an ideal model organism for studying climate-driven changes in species distribution patterns. However, its capacity to track shifting climatic niches is likely constrained by its reproductive biology. *D. macropodum* is a dioecious species and relies primarily on gravity and short-distance bird dispersal for spreading its relatively large seeds [23]. This limited dispersal potential, coupled with habitat fragmentation, may severely restrict its natural migration rate, increasing its vulnerability to rapid climate change [24].

Although *D. macropodum* has been studied from taxonomic, phytochemical, and eco-physiological perspectives [23,25,26], a systematic and comprehensive analysis of its national-scale distribution patterns, environmental drivers, and dynamic responses to future climate change has not yet been conducted. Species distribution models (SDMs), particularly the Maximum Entropy (MaxEnt) model, have served as powerful tools for quantifying the relationships between species and their environment [27,28]. Among these, the Maximum Entropy (MaxEnt) framework was chosen for this study. While ensemble platforms (e.g., BIOMOD2) and process-based mechanistic models offer valuable alternative approaches, MaxEnt was selected as the most appropriate tool due to its proven high performance with presence-only data, computational efficiency for high-resolution national-scale projections, and robust interpretability of species–environment relationships [29]. However, several critical limitations in current research have been identified: (1) default model parameters are often used directly, while the essential role of parameter optimization in controlling overfitting and improving model transferability is frequently neglected [30,31]; (2) the selection of environmental variables is mostly confined to bioclimatic factors [32,33,34], which may overlook important non-climatic drivers; and (3) species dispersal limitations are generally not incorporated into future projections, resulting in overly idealized predictions of potential habitats [35,36], as the model assumes unlimited migration capability. Our parameter optimization framework using ENMeval further ensures that model complexity is rigorously controlled, addressing a key concern that is often mitigated by ensemble techniques [37].

Therefore, this study proposes an integrated analytical framework designed to achieve four main objectives. First, we aim to precisely identify the dominant environmental factors that determine the contemporary distribution of *D. macropodum* and to quantify its niche requirements. Second, a high-resolution map of the species’ current suitable habitat is generated. Third, changes in habitat area, spatial shift trajectories, and fragmentation degree are projected under multiple climate scenarios, including SSP126, SSP245, and SSP585, across different time periods, including the 2050s, 2070s, and 2090s. Finally, by classifying areas into stability, contraction, and expansion zones and integrating centroid shift analysis, we quantitatively assess the impacts of climate change [38]. This helps to identify key climate refugia and assisted-migration target areas, providing spatially explicit scientific support for conservation planning. Based on this framework, four testable hypotheses (H) are proposed. H1 states that the predictive accuracy of the parameter-optimized MaxEnt model, which uses grid search of regularization multipliers and feature classes implemented with the ENMeval package, will be significantly higher than that of the default parameter model. This will be primarily measured by the AUC metric and accompanied by improved transferability and robustness. H2 proposes that the current distribution of *D. macropodum* is jointly controlled by moisture and temperature seasonality factors, for example, Bio19, Bio2, and Bio18, and that the cumulative contribution of these factors will be significantly higher than that of other environmental variables. H3 anticipates that under future climate scenarios, the suitable habitat area of *D. macropodum* will decrease significantly as greenhouse gas concentrations increase. We hypothesize that the core distribution will shift to track suitable climatic conditions, which may manifest as migration toward regions with buffered microclimates (e.g., higher elevations or topographically complex areas) rather than a simple latitudinal shift. This process is expected to be accompanied by intensified habitat fragmentation and potential geographic redistribution, with the most severe impacts under the high-forcing scenario, SSP585. H4 predicts that the centroid of suitable habitat will undergo significant spatial displacement, showing a general movement trajectory toward the southwest or southeast. The migration distance will increase under higher-emission scenarios, reflecting an adaptive response of the species to track its optimal climatic conditions. Therefore, employing an optimized MaxEnt model with rigorously selected variables, this study projects future range shifts for *D. macropodum* under climate change. Ultimately, the proposed framework not only tests specific hypotheses concerning its climatic drivers and spatial redistribution but also provides a transferable methodology for forecasting climatic impacts on other evergreen tree species.

## 2. Materials and Methods

### 2.1. Occurrence Data Acquisition and Processing

**Figure 1:**
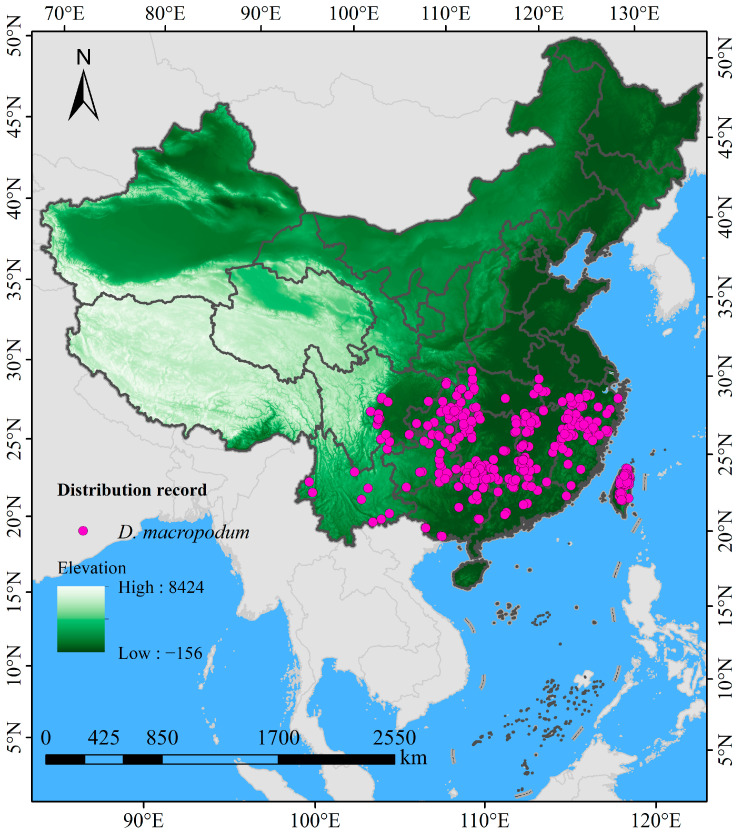
Occurrence locations of *Daphniphyllum macropodum* across China.

The occurrence data of *D. macropodum* within China were aggregated from several openly accessible repositories and scholarly sources, including the Chinese Virtual Herbarium (CVH, https://www.cvh.ac.cn/, accessed 12 March 2025), the Global Biodiversity Information Facility (GBIF, https://doi.org/10.15468/dl.vz8azj, accessed 12 March 2025) [39], the China National Knowledge Infrastructure (CNKI, https://www.cnki.net/, accessed 15 March 2025), Web of Science (https://www.webofscience.com/, accessed 15 March 2025), and Google Scholar (https://ac.scmor.com/, accessed 18 March 2025). In cases where records included detailed collection site information (e.g., town or village) but were missing the exact latitude and longitude, the corresponding coordinates were acquired through the Jingweidu Query Positioning Tool (http://jingweidu.757dy.com/, accessed 28 May 2025). Redundant entries were eliminated via the “Remove Duplicates” feature in Excel 2016, producing a preliminary total of 480 records. To reduce the effects of sampling bias and spatial overrepresentation [31], a 2.5′ grid was generated using ArcGIS 10.8, within which only the occurrence point nearest to the center of each grid cell was preserved. The 2.5 arcminute (~5 km at the equator) grid cell size was selected to align with the resolution of the primary environmental variables (WorldClim bioclimatic layers), thereby minimizing spatial autocorrelation while preserving sufficient environmental heterogeneity for model training [40]. This manual grid-based thinning approach is functionally analogous to and produces results consistent with commonly used algorithmic methods such as the “spThin” R package [41]. To address potential temporal bias arising from occurrence records collected over multiple decades, we implemented a stringent temporal filtering protocol. Only records from after the year 1970 were retained, ensuring that the species occurrence data closely corresponded to the temporal baseline of the climate normal (1970–2000) used for model calibration. Older records, which might represent historical populations under divergent climatic conditions, were excluded from the analysis to prevent introducing mismatch between species presence and contemporary climate layers. This filtering step resulted in a refined dataset comprising 354 distribution records (Figure 1) suitable for subsequent modeling. All data were prepared and saved in CSV format, following the input specifications of MaxEnt software version 3.4.4 (http://biodiversityinformatics.amnh.org/open_source/maxent/, accessed 22 March 2025). The 1:10,000,000 administrative basemap of China was acquired from the Ministry of Natural Resources (GS (2023)2762; http://bzdt.ch.mnr.gov.cn, accessed on 22 December 2023), georeferenced in ArcGIS 10.8, and vectorized into a Shapefile following the GCS_WGS_1984 geographic coordinate system.

### 2.2. Acquisition and Processing of Environmental Variables

In this study, 24 environmental variables (Table S1), encompassing climatic, topographic, vegetation, and anthropogenic dimensions, were systematically selected to predict the distribution pattern of *D. macropodum*. Among these, 19 bioclimatic variables and elevation data were obtained from WorldClim 2.1 (https://www.worldclim.org/, accessed on 8 March 2025). Slope and aspect were derived from the elevation data using ArcGIS 10.8. Vegetation cover was characterized using the Normalized Difference Vegetation Index (NDVI) based on the mean annual value derived from the MOD13A3 product (2000–2022 period) [42], while human activity intensity was quantified using the Human Footprint Index [43]. To evaluate the potential impacts of future climate change on the distribution of *D. macropodum*, the BCC-CSM2-MR climate model, which has demonstrated good performance in simulating East Asian climates [44], was selected. Projections under three Shared Socioeconomic Pathways—SSP126 (low forcing, ≈1.8 °C warming by 2100), SSP370 (intermediate forcing, ≈2.7 °C), and SSP585 (high forcing, ≈4.4 °C) [45]—were used to analyze distribution changes across three periods: the 2050s (2041–2060), 2070s (2061–2080), and 2090s (2081–2100).

To reduce the impact of multicollinearity, Spearman correlation analysis (Figure S1) and variance inflation factor (VIF) testing were applied to all initial environmental variables across the entire study area background, using the usdm package in R version 4.2.2. The variable selection followed a stepwise process: variables with VIF ≥ 10 were removed first; for remaining variables with |r| ≥ 0.7, the variable with greater ecological relevance to *D. macropodum* was retained. This procedure adhered to best practices for addressing multicollinearity [46]. This screening procedure resulted in the identification of eleven environmental variables for subsequent modeling, comprising six climatic, three topographic, one anthropogenic, and one vegetation-related variable (Table 1).

### 2.3. MaxEnt Model Optimization and Prediction

#### 2.3.1. MaxEnt Model Optimization

To mitigate the risk of systematic errors and mismatch between forecasts and empirical observations [31], we employed the ENMeval 2.0.4 R package [47] for automated parameter optimization and calibration. This tool addresses overfitting by systematically evaluating model complexity through a grid search across biologically meaningful parameter combinations. Specifically, the ENMevaluate function tests a predefined range of regularization multiplier (RM) values from 0.5 to 4.0 (in increments of 0.5), which control the penalty for model complexity, alongside nine distinct feature class (FC) combinations (e.g., L, H, LQ, HPT, LQH, QHP, QHPT, LQHPT). These FCs are derived from five feature types: linear (L), quadratic (Q), product (P), threshold (T), and hinge (H), which together determine the shape of species–environment response curves [48]. For each combination, the algorithm fits a model and computes the corrected Akaike Information Criterion (AICc), which balances goodness-of-fit and complexity. The optimal parameter set (RM = 4, FC = QHPT) was selected solely based on the lowest AICc value, indicating the most parsimonious model with the strongest predictive performance while minimizing overfitting [37,49]. This objective, data-driven tuning process significantly enhances the ecological realism and transferability of our habitat projections for *D. macropodum* under climate change scenarios.

#### 2.3.2. Parameter Configuration of the MaxEnt Model

To elucidate the potential suitable habitat pattern of *D. macropodum* under global climate change, a MaxEnt species distribution modeling workflow that balances accuracy and robustness was developed based on 354 current distribution records that underwent strict quality control. The occurrence data were first partitioned using stratified random sampling into a 75% training set (266 records) and a 25% independent validation set (89 records), thereby preserving the structural representativeness of the environmental space and ensuring statistical independence in model evaluation. Key parameters were then systematically optimized, following best practices in contemporary ecological niche modeling: a cross-validation strategy was adopted to enhance internal validation; 10,000 background points were configured to adequately capture environmental heterogeneity; the regularization multiplier was set to 4 to mitigate overfitting risks; and the feature class combination was specified as QHPT (quadratic, hinge, product, threshold) to more accurately capture complex non-linear responses of the species to environmental drivers. The model was run repeatedly for 10 iterations and the results were ensemble-averaged to reduce uncertainty arising from stochastic variation. The final output was logistic-formatted raster data representing habitat suitability probability, which could be directly used for subsequent spatial statistical analysis, multi-scenario comparison, and visualization.

#### 2.3.3. Accuracy Assessment of the Optimized Model Predictions

The predictive performance of the MaxEnt model was comprehensively evaluated using three metrics: the Area Under the Curve (AUC) of the Receiver Operating Characteristic (ROC) curve, the True Skill Statistic (TSS), and Cohen’s kappa coefficient (Kappa) [50]. The AUC value, which ranges from 0.5 to 1, was employed to measure the model’s ability to distinguish presence points from background points at all thresholds [48]. According to widely accepted criteria, AUC values of 0.5–0.6 indicate no discriminatory capacity, values of 0.6–0.7 indicate low accuracy, values of 0.7–0.8 indicate moderate accuracy, values of 0.8–0.9 indicate high accuracy, and values of 0.9–1.0 indicate very high predictive accuracy [51]. The TSS, which combines sensitivity and specificity to correct for sample bias, was interpreted as follows: values below 0.4 indicate poor performance, values of 0.4–0.8 indicate good performance, and values above 0.8 indicate excellent performance [52]. The Kappa coefficient was applied to quantify agreement between model predictions and observed distributions while accounting for random chance; values below 0.4 were considered poor, values of 0.4–0.75 were considered good, and values above 0.75 indicated excellent agreement [53].

### 2.4. Classification of Potentially Suitable Areas for the Species

To enhance the ecological interpretability and spatial mapping precision of model predictions, the MaxEnt model was run with 10 replicate cross-validations, and the “Maximum Training Sensitivity Plus Specificity” (MTSPS) logistic threshold was selected as the criterion for discriminating suitable from non-suitable habitats [54]. Specifically, the mean MTSPS threshold across the 10 replicates was calculated and applied as a unified cut-off value for binary classification, followed by reclassification and visualization of the habitat suitability probability raster for *D. macropodum*. The MTSPS method is widely recommended in ecological niche modeling due to its low sensitivity to variations in species prevalence and background point selection [55,56,57,58]; its strong robustness helps reduce omission errors for species with low prevalence while mitigating overfitting risks for widespread species, an advantage that has been validated by Liu et al. [59]. After obtaining the continuous suitability probability surface from MaxEnt, the results were imported into ArcGIS 10.8, where the “Reclassify” tool was used, in conjunction with the Natural Breaks (Jenks) method, for classification [60,61]. This method objectively identifies inherent breakpoints in the probability distribution by maximizing within-class homogeneity and between-class heterogeneity, thereby delineating suitability grade boundaries without subjective bias. The potential suitable habitat was ultimately classified into four consecutive grades: unsuitable (0–0.140), generally suitable (0.140–0.3), moderately suitable (0.3–0.5), and highly suitable (0.5–1.0).

### 2.5. Spatial Pattern Changes in the Suitable Habitat for D. macropodum

Under global climate change, potential species suitability is categorized into three core categories based on spatiotemporal dynamics: stable zones (areas suitable in both current and future periods), contraction zones (currently suitable but projected to become unsuitable), and expansion zones (currently unsuitable but projected to become suitable). This classification framework enables systematic assessment of species’ distribution responses to climatic drivers and provides a scientific basis for identifying priority conservation areas and formulating adaptive strategies. To evaluate the spatial shifts in the potential suitability of *D. macropodum* across future climate scenarios and time periods, a series of spatial analyses were conducted in ArcGIS 10.8 based on habitat suitability probability outputs from the MaxEnt model. The current and future suitability outputs generated by the model, including projections for the 2050s, 2070s, and 2090s under the SSP126, SSP245, and SSP585 scenarios, were imported into ArcGIS. Using the MTSPS threshold of 0.140 as a binary threshold, continuous probability surfaces were converted into binary distribution maps, where retention was encoded as 1→1, contraction as 1→0, and expansion as 0→1 [62], thereby clearly distinguishing suitable from non-suitable areas. The binary raster data for each period were then converted into vector polygon layers to support subsequent overlay operations. Finally, using the “Intersect” tool in ArcGIS, future suitability vectors were spatially overlaid with current suitability distributions to generate maps illustrating changes in the spatial pattern of suitable habitats for *D. macropodum* under different climate scenarios.

### 2.6. Centroid Shift in Suitable Habitat for D. macropodum

The centroid locations of potential suitable habitats for *D. macropodum* were extracted with SDMtoolbox v2.0 [63], and their spatial migration distances under multiple climate change scenarios were systematically quantified. The study periods encompassed the current climatic baseline (1970–2020) and three future periods (2050s, 2070s, and 2090s), while three Shared Socioeconomic Pathways (SSP126, SSP370, and SSP585) were incorporated to evaluate the dynamic trajectories of the species’ range centroids. The continuous habitat suitability probability raster generated by the MaxEnt model was first imported into ArcGIS 10.8, binarized with a threshold of 0.140 to distinguish suitable from non-suitable area. The geometric statistics module within the spatial analysis toolbox was then employed to calculate the centroid of suitable habitat polygons for each period and scenario by selecting “Centroid” as the geometric representation type. The longitude and latitude coordinates were then accurately extracted. Finally, based on centroid coordinate shifts across different periods and scenarios, the Euclidean distance formula was used to quantify shift distances of the habitat centroid at various spatiotemporal scales, thereby quantitatively revealing the potential spatial displacement characteristics of this species in response to climate change. To mitigate potential edge effects caused by habitat fragmentation, the centroid was calculated based on the geometry of the entire suitable habitat distribution, rather than on fragmented patches. This approach provides a macroscopic summary of the range’s center of mass, while acknowledging that fine-scale fragmentation may influence local outcomes.

## 3. Results

### 3.1. Optimization and Accuracy Evaluation of the MaxEnt Model

**Figure 2:**
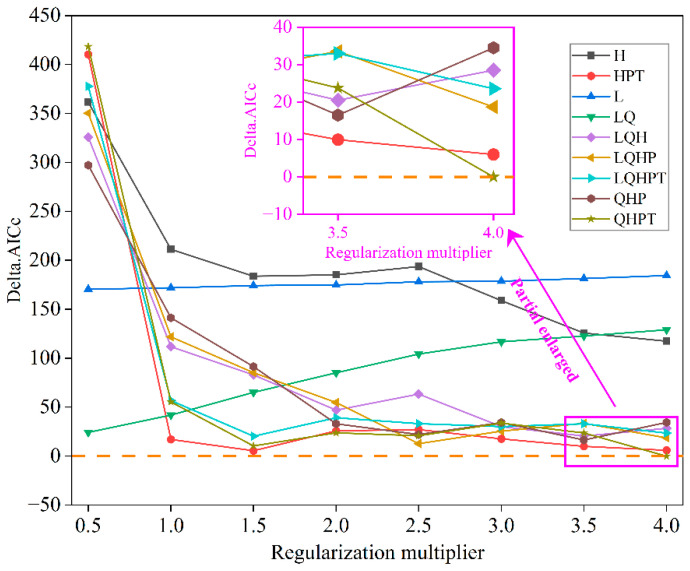
Variation in Delta AICc values obtained from ENMeval-based model selection for *Daphniphyllum macropodum*. Feature classes are denoted as follows: L (linear), Q (quadratic), H (hinge), P (product), T (threshold). Horizontal orange dashed line marks zero reference level for Delta AICc.

Delta AICc served as the core metric for parameter optimization of the MaxEnt model. The initial default parameters (RM = 1, FC = LQHP) produced a high Delta AICc value of 121.87 (Figure 2), greatly exceeding the recommended threshold (ΔAICc ≤ 2), suggesting substantial overfitting and model uncertainty. To enhance robustness, we conducted a systematic grid search using the ENMeval package (v2.0.4), incorporating 354 occurrence records and 11 environmental variables. The search evaluated regularization multipliers from 0.5 to 4.0 and multiple feature class combinations (L, Q, H, P, T). The parameter set with RM = 4 and FC = QHPT was selected under AICc optimization, achieving a Delta AICc of 0, indicating optimal fit, improved predictive reliability, and minimal overfitting.

**Figure 3:**
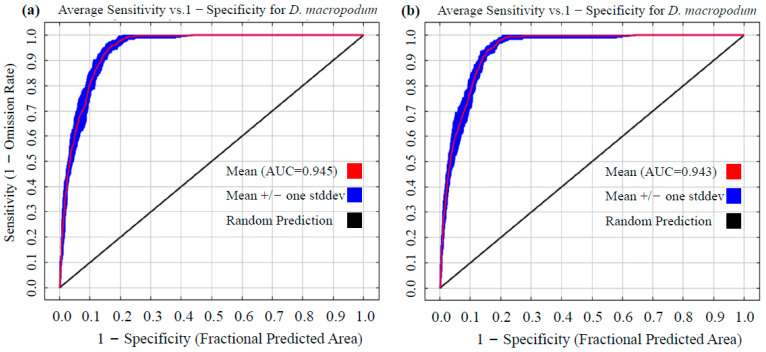
Evaluation of prediction accuracy for *Daphniphyllum macropodum* based on Receiver Operating Characteristic (ROC) curve in MaxEnt modeling: (**a**) Area Under the Curve (AUC) value obtained using default parameters; (**b**) AUC value achieved after parameter optimization.

A further comparison of modeling performance between the default (RM = 1, FC = LQHP) and optimized (RM = 4, FC = QHPT) parameter sets was conducted. Based on ensemble results from 10 replicate cross-validations, the AUC value was 0.945 under default parameters (Figure 3a) and 0.943 under optimized parameters (Figure 3b). Both values significantly exceed the “excellent” model discrimination threshold (AUC ≥ 0.9). Additionally, the optimized model achieved a True Skill Statistic (TSS) of 0.81 and a Kappa coefficient of 0.50, indicating excellent and good performance, respectively, according to established thresholds. This confirms that the optimized model retained high explanatory power for species–environment relationships. Although a minor decrease in the AUC was observed after optimization (a reduction of 0.21%, ΔAUC = −0.002), overfitting caused by the default parameters was effectively suppressed through enhanced regularization and simplified feature classes. This multi-metric validation approach provides a more reliable modeling framework for predicting species suitability.

### 3.2. Factors Influencing the Distribution of D. macropodum in China

**Figure 4:**
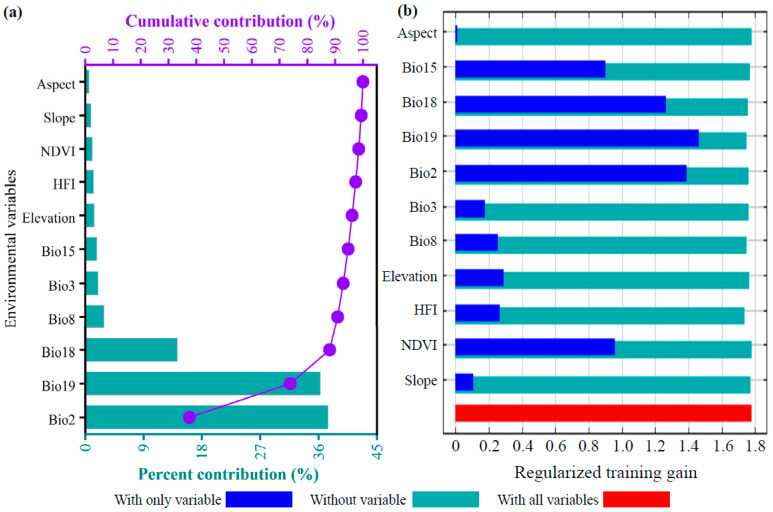
Environmental variable importance assessment for *Daphniphyllum macropodum* based on MaxEnt modeling. (**a**) Percentage contribution of each variable; (**b**) jackknife test of regularized training gain.

Based on the optimized MaxEnt model, the influences of 11 environmental factors on the potential distribution of *D. macropodum* were systematically evaluated using both variable contribution rates and jackknife test results. The environmental variables are listed in descending order of contribution (Figure 4a): mean diurnal range (Bio2, 37.5%), precipitation of coldest quarter (Bio19, 36.3%), precipitation of warmest quarter (Bio18, 14.2%), mean temperature of wettest quarter (Bio8, 2.9%), isothermality (Bio3, 2.0%), precipitation seasonality (Bio15, 1.8%), elevation (1.4%), Human Footprint Index (HFI, 1.3%), NDVI (1.1%), slope (0.9%), and aspect (0.6%). By category, climatic factors collectively accounted for 94.7% of the total contribution, followed by topographic factors at 2.9%, human activity factors at 1.3%, and vegetation factors (NDVI) at 1.1%. The jackknife test of regularized training gain (Figure 4b) revealed that the top seven factors with the highest gain under the “only one variable used” mode were Bio19, Bio2, Bio18, Bio15, NDVI, elevation, and HFI. Based on the conventional threshold of cumulative contribution exceeding 85% [64], Bio2, Bio19, and Bio18 together explained 88.0% of the variation in the suitable habitat distribution. These three factors overwhelmingly dominated the geographic pattern of this species within China, and were thus identified as the core climatic drivers decisively shaping its potential distribution.

**Figure 5:**
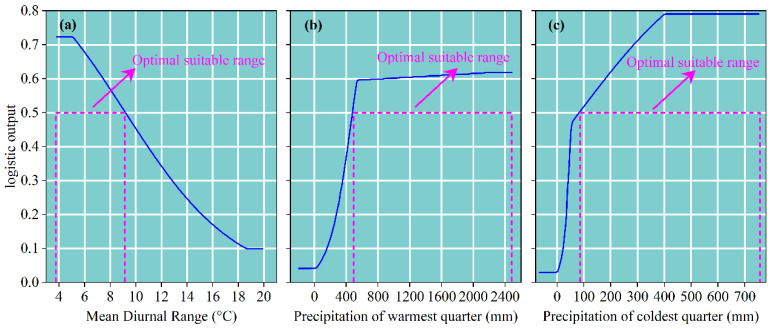
Response curves depicting the relationship between environmental variables and the predicted presence probability of *D. macropodum*. The blue curves represent the mean values derived from 10 replicate model runs, while the purple dashed lines indicate the optimal range of suitability. (**a**) Mean diurnal range; (**b**) precipitation of warmest quarter; (**c**) precipitation of coldest quarter.

To clarify the relationship between the presence probability of *D. macropodum* and three key environmental variables, single-variable response curves for the dominant factors were plotted (Figure 5). The analysis showed that the presence probability remained stable at 0.72 when the mean diurnal range (Bio2) ranged between 3.81 °C and 5.02 °C. As Bio2 increased from 5.03 °C to 18.46 °C, the presence probability decreased sharply from 0.71 to 0.10, and stabilized around 0.10 beyond 18.46 °C (Figure 5a). For the precipitation of the coldest quarter (Bio19), the presence probability remained consistently low (0.04) within the range of −198.12 mm to 28.15 mm. It increased rapidly from 0.04 to 0.59 as Bio19 rose from 28.16 mm to 542.91 mm, and then gradually reached a peak of 0.62 within the interval of 542.92 mm to 2483.22 mm (Figure 5b). Regarding the precipitation of the warmest quarter (Bio18), the probability stayed constant at 0.03 from −58.86 mm to −4.15 mm, rose sharply to 0.47 when Bio18 increased from −4.16 mm to 57.39 mm, and then increased further to 0.78 within the range of 57.40 mm to 399.32 mm, before stabilizing at 0.79 beyond 399.33 mm (Figure 5c). According to commonly used suitability thresholds in niche modeling, environmental ranges corresponding to a presence probability of no less than 0.5 are considered to reflect optimal habitat conditions [65]. Thus, the most suitable environmental ranges for *D. macropodum* were determined as follows: Bio2 between 3.80 °C and 9.18 °C, Bio19 between 484.65 mm and 2483.22 mm, and Bio18 between 91.59 mm and 753.22 mm (Figure 5).

### 3.3. Potential Suitable Distribution of D. macropodum Under Current Climatic Conditions in China

**Figure 6:**
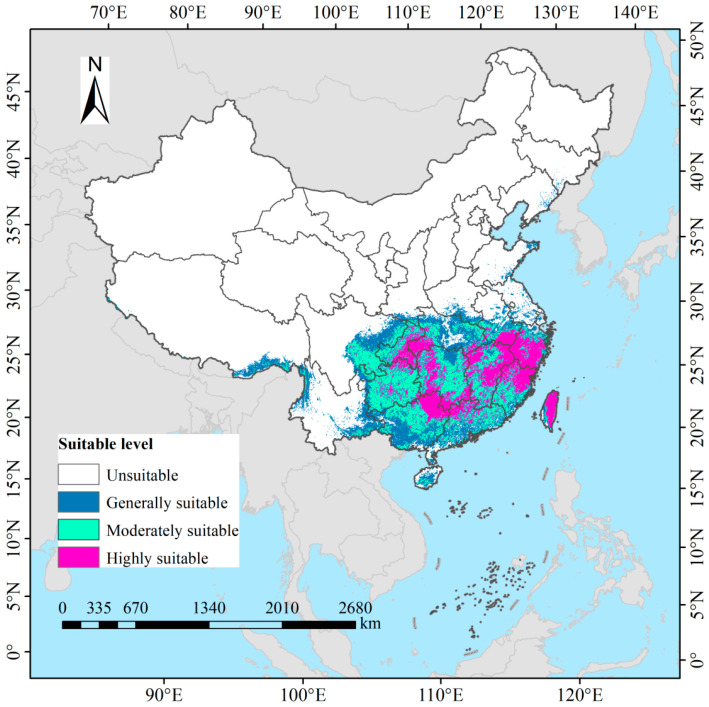
Modeled current climatically suitable habitats for *Daphniphyllum macropodum* across China.

Under current climatic conditions, the total potentially suitable habitat area for *D. macropodum* in China is estimated at approximately 167.47 × 10^4^ km^2^, representing 17.44% of the national land area (Figure 6). Highly suitable areas, covering 51.53 × 10^4^ km^2^ (5.37%), are primarily distributed across Taiwan Province, Zhejiang Province, northern Fujian Province, northern and eastern Jiangxi Province, southern Anhui Province, northern Guangdong Province, the Guangxi Zhuang Autonomous Region, western and southern Hunan Province, western Hubei Province, the southeastern Chongqing Municipality, and central and northern Guizhou Province. Moderately suitable areas extend over 66.39 × 10^4^ km^2^ (6.92%), with major concentrations in most parts of Guizhou Province, southeastern Sichuan Province, western Chongqing Municipality, central Guangxi Zhuang Autonomous Region, eastern and western Hubei Province, eastern Hunan Province, southern Jiangxi Province, and southern Fujian Province. Low-suitability regions span 49.55 × 10^4^ km^2^ (5.16%) and are broadly scattered throughout Hainan Province, southeastern Xizang Autonomous Region, eastern and western Yunnan Province, northeastern Sichuan Province, southern Guangxi Zhuang Autonomous Region, southern Guangdong Province, central Hubei Province, and northern Hunan Province.

### 3.4. Projected Distribution of D. macropodum in China Under Future Climate Scenarios

**Figure 7:**
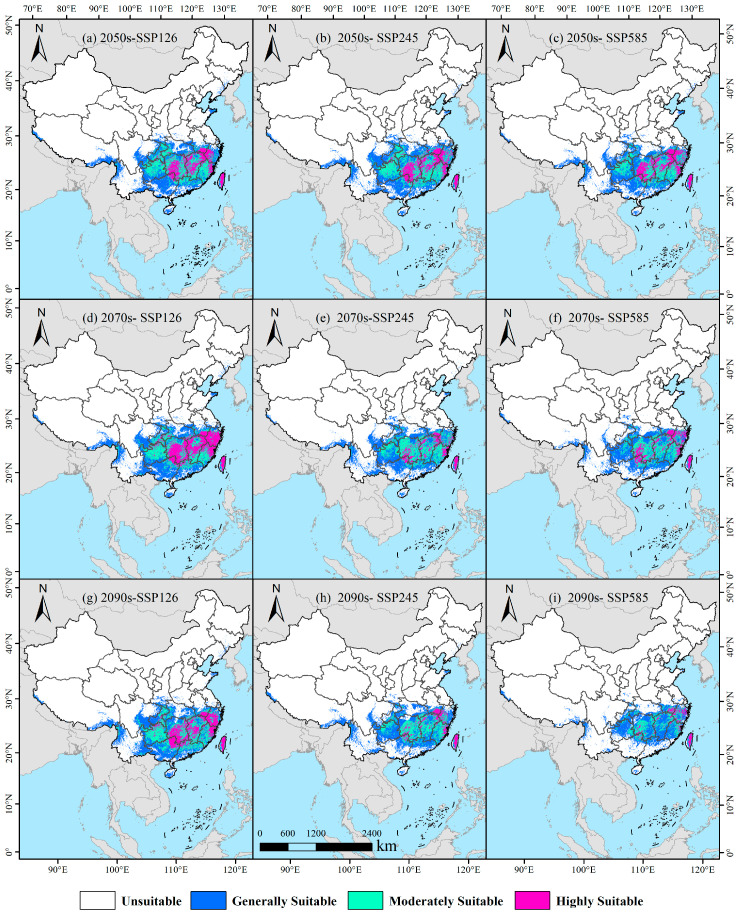
Modeled distribution of *Daphniphyllum macropodum* across China in response to projected climate change.

**Figure 8:**
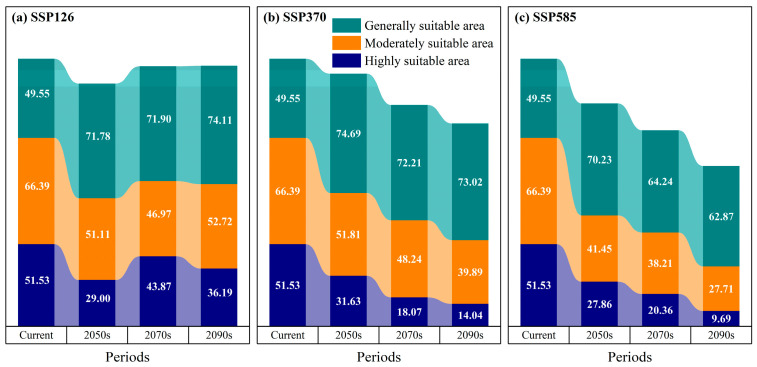
Projected changes in suitable habitat area for *Daphniphyllum macropodum* under various climate scenarios (unit: 10^4^ km^2^).

Under the SSP126 climate scenario, projections indicate a generally stable to slightly expanding distribution of potentially suitable habitat for *D. macropodum* in China throughout the mid-to-late 21st century (Figure 7). By the 2050s, the total suitable area is estimated to reach 151.88 × 10^4^ km^2^, representing 15.82% of the national land area (Figure 8). This area was subdivided into low- (71.78 × 10^4^ km^2^), medium- (51.11 × 10^4^ km^2^), and high-suitability regions (29.00 × 10^4^ km^2^). Spatially, high-suitability zones are predominantly located in Taiwan Province, northeastern Fujian Province, southwestern Zhejiang Province, southern Anhui Province, central Jiangxi Province, central Hunan Province, and the southern Guangxi Zhuang Autonomous Region (Figure 7a). By the 2070s, the total suitable habitat area is projected to increase to 162.74 × 10^4^ km^2^, accounting for 16.95% of China’s land area. The areal extent of low-, medium-, and high-suitability regions is recorded as 71.90 × 10^4^ km^2^, 46.97 × 10^4^ km^2^, and 43.87 × 10^4^ km^2^, respectively. The spatial distribution of high-suitability areas during this period remains largely consistent with the 2050s pattern, maintaining a strong presence in Taiwan Province, Zhejiang Province, northeastern Fujian Province, southern Anhui Province, central Jiangxi Province, central Hunan Province, and southern Guangxi Zhuang Autonomous Region (Figure 7d). In the 2090s, the total suitable area is projected to further expand to 163.02 × 10^4^ km^2^, constituting 16.98% of the national territory. The corresponding areas for low-, medium-, and high-suitability regions measure 74.11 × 10^4^ km^2^, 52.72 × 10^4^ km^2^, and 36.19 × 10^4^ km^2^, respectively. The distribution of high-suitability zones shows moderate expansion compared to previous periods, with new areas emerging in northern Guangdong Province, while a consistent presence is maintained in Taiwan Province, Zhejiang Province, northeastern Fujian Province, southern Anhui Province, central Jiangxi Province, central Hunan Province, and southern Guangxi Zhuang Autonomous Region (Figure 7g).

Under the SSP245 climate scenario, a consistent decline is projected for the total potentially suitable habitat area of *D. macropodum* in China from the mid-to-late 21st century (Figure 7). In the 2050s (2041–2060), the total suitable area is projected to reach approximately 158.13 × 10^4^ km^2^, constituting 16.47% of the national land area (Figure 8). This distribution includes low-, medium-, and high-suitability regions covering 74.69 × 10^4^ km^2^, 51.81 × 10^4^ km^2^, and 31.63 × 10^4^ km^2^, respectively. Geographically, high-suitability zones are predominantly identified in Taiwan Province, northeastern Fujian Province, southwestern Zhejiang Province, southern Anhui Province, central Jiangxi Province, central Hunan Province, and southern Guangxi Zhuang Autonomous Region (Figure 7b). By the 2070s, the total suitable area is expected to diminish to 138.52 × 10^4^ km^2^, equivalent to 14.43% of China’s terrestrial extent. The areal coverage of low-, medium-, and high-suitability regions is recorded as 72.21 × 10^4^ km^2^, 48.24 × 10^4^ km^2^, and 18.07 × 10^4^ km^2^, respectively. A discernible spatial shift emerges in the high-suitability areas, which are expected to become increasingly concentrated within Taiwan Province, northeastern Fujian Province, central and eastern Jiangxi Province, southern Anhui Province, southwestern Hunan Province, and northeastern Guangxi Zhuang Autonomous Region (Figure 7e). Towards the end of the century (2090s), the total suitable habitat it expected to contract further to 126.96 × 10^4^ km^2^, representing merely 13.23% of the national land area. The corresponding areas for low-, medium-, and high-suitability categories are 73.02 × 10^4^ km^2^, 39.89 × 10^4^ km^2^, and 14.04 × 10^4^ km^2^, respectively. During this period, high-suitability regions will undergo substantial reduction and fragmentation, persisting only in limited portions of Taiwan Province, southern Anhui Province, and western Zhejiang Province (Figure 7h).

Under the high-emission SSP585 scenario, a progressive reduction in potentially suitable habitat for *D. macropodum* across China is projected throughout the mid-to-late 21st century (Figure 7). By the 2050s (2041–2060), the total suitable area is projected to reach approximately 139.54 × 10^4^ km^2^, representing 14.54% of China’s total land area (Figure 8). This area comprises low-, medium-, and high-suitability regions covering 70.23 × 10^4^ km^2^, 41.45 × 10^4^ km^2^, and 27.86 × 10^4^ km^2^, respectively. High-suitability zones form a relatively continuous distribution, primarily encompassing Taiwan Province, northeastern Fujian Province, western Zhejiang Province, southern Anhui Province, central Jiangxi Province, central Hunan Province, and parts of southern Guangxi Zhuang Autonomous Region (Figure 7c). Toward the 2070s (2061–2080), the total suitable area is expected to further decline to 122.81 × 10^4^ km^2^, accounting for 12.79% of the national territory. The areal extent of low-, medium-, and high-suitability regions measures 64.24 × 10^4^ km^2^, 38.21 × 10^4^ km^2^, and 20.36 × 10^4^ km^2^, respectively. Concurrently, the high-suitability areas exhibit increased fragmentation and are mainly confined to Taiwan Province, northeastern Fujian Province, central and western Zhejiang Province, southern Anhui Province, western Hunan Province, and southern Guangxi Zhuang Autonomous Region (Figure 7f). By the 2090s (2081–2100), a pronounced contraction is projected to occur, with the total suitable habitat diminishing to 100.26 × 10^4^ km^2^—only 10.44% of China’s land area. The coverage of low-, medium-, and high-suitability regions will decrease to 62.87 × 10^4^ km^2^, 27.71 × 10^4^ km^2^, and 9.69 × 10^4^ km^2^, respectively. High-suitability areas are expected to undergo severe reduction and fragmentation, persisting only in scattered sections of Taiwan Province, central and northern Zhejiang Province, and southern Anhui Province (Figure 7i).

### 3.5. Changes in Suitable Habitat Area for D. macropodum Under Future Climate Scenarios

**Figure 9:**
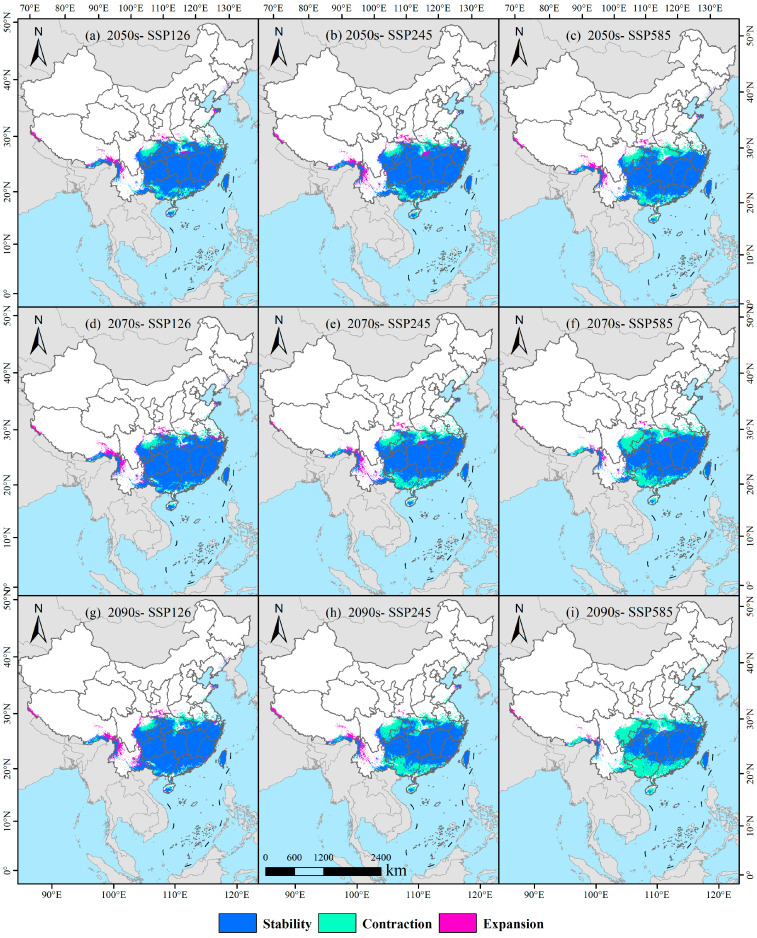
Projected patterns of habitat stability, contraction, and expansion for *Daphniphyllum macropodum* under future climate change.

Under the SSP126 climate scenario, distinct temporal shifts are observed in the spatial distribution of potentially suitable habitat for *D. macropodum* (Figure 9; Table 2). In the 2050s, the retained habitat area is projected to reach 173.41 × 10^4^ km^2^, accounting for 80.65% of the original suitable habitat, while the lost and expanded areas are expected to measure 30.27 × 10^4^ km^2^ (14.08%) and 11.35 × 10^4^ km^2^ (5.28%), respectively (Figure 9a). By the 2070s, the retained area will increase to 182.01 × 10^4^ km^2^ (82.80%), with a corresponding decrease in lost area to 21.71 × 10^4^ km^2^ (9.88%) and an increase in expanded area to 16.09 × 10^4^ km^2^ (7.32%) (Figure 9d). In the 2090s, a slight reduction is projected to occur in the retained area, which is expected to decline to 181.88 × 10^4^ km^2^ (82.56%), whereas the lost area shows a minor increase to 21.83 × 10^4^ km^2^ (9.91%). Meanwhile, the expanded area will continue to grow, reaching 16.60 × 10^4^ km^2^ (7.54%) (Figure 9g).

Under the SSP245 scenario, a clear temporal progression emerges in habitat pattern changes (Figure 9; Table 2). In the 2050s, the retained area is expected to amount to 176.83 × 10^4^ km^2^ (80.64%), accompanied by lost and expanded areas of 26.86 × 10^4^ km^2^ (12.25%) and 15.58 × 10^4^ km^2^ (7.11%), respectively (Figure 9b). By the 2070s, the retained area is projected to decrease to 157.69 × 10^4^ km^2^ (73.51%), alongside a notable rise in lost area to 46.01 × 10^4^ km^2^ (21.45%) and a contraction in expanded area to 10.83 × 10^4^ km^2^ (5.05%) (Figure 9e). This trend is expected to persist into the 2090s, with the retained area further declining to 144.23 × 10^4^ km^2^ (67.51%), while the lost area will increase to 59.41 × 10^4^ km^2^ (27.81%) and the expanded area will diminish to 10.00 × 10^4^ km^2^ (4.68%) (Figure 9h).

Under the high-emission SSP585 scenario, more pronounced habitat alterations are projected (Figure 9; Table 2). The 2050s are expected to be characterized by a retained area of 159.18 × 10^4^ km^2^ (74.35%), with 44.50 × 10^4^ km^2^ lost (20.79%) and 10.42 × 10^4^ km^2^ expanded (4.87%) (Figure 9c). By the 2070s, the retained area is expected to diminish to 142.34 × 10^4^ km^2^ (67.59%), whereas the lost area will increase substantially to 61.32 × 10^4^ km^2^ (29.12%) and the expanded area will decline to 6.92 × 10^4^ km^2^ (3.29%) (Figure 9f). The most substantial changes are projected for the 2090s, as the retained area is expected to contract sharply to 117.81 × 10^4^ km^2^ (56.78%) and the lost area to escalate to 85.83 × 10^4^ km^2^ (41.36%). Concurrently, the expanded area is projected to decrease to its lowest value of 3.85 × 10^4^ km^2^, representing a minimal expansion rate of only 1.86% (Figure 9i).

### 3.6. Centroid Shift for D. macropodum Suitable Habitat Under Multiple Climate Scenarios

**Figure 10:**
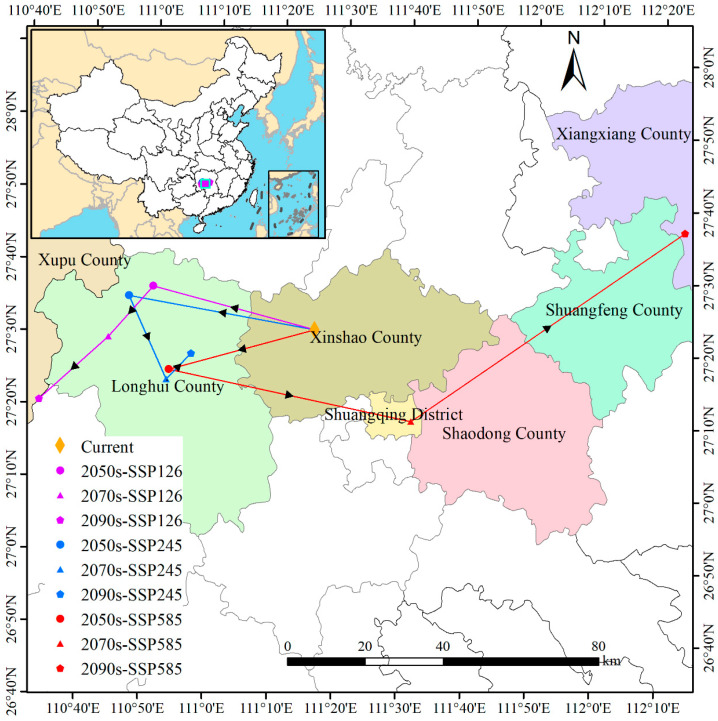
Shifts in the spatial centroid of climatically suitable habitat for *Daphniphyllum macropodum* under different climate scenarios.

Under current climatic conditions, the centroid of suitable habitat for *D. macropodum* is located in Daxin Town, Xinshao County, Hunan Province (111°21′ E, 27°28′ N). Projections under future climate scenarios indicate that the distribution centroid will undergo substantial spatial shifts, exhibiting complex spatiotemporal dynamics (Figure 10). Specifically, under the SSP126 scenario, the centroid demonstrates a persistent southwestern trajectory: by the 2050s, it shifts 43.24 km northwest to Jinshiqiao Town, Longhui County (110°56′ E, 27°35′ N); subsequently, it moves an additional 17.38 km southwest to Dashuitian Township (110°49′ E, 27°28′ N) by the 2070s; and finally, it displaces a further 23.94 km southwest to Longtan Town, Xupu County (110°37′ E, 27°20′ N), by the 2090s. In contrast, the SSP245 scenario reveals a non-linear and reversing migration pattern. The centroid first moves 48.75 km northwest by the 2050s to Jinshiqiao Town (110°52′ E, 27°34′ N), then shifts 23.40 km southeast to Liuduzhai Town (110°57′ E, 27°22′ N) by the 2070s, and ultimately relocates 9.19 km northeast to Qijiang Town (111°02′ E, 27°25′ N) by the 2090s. Under the high-emission SSP585 scenario, more extreme and variable displacements are projected. The centroid shifts 39.11 km southwest to Liuduzhai Town (110°58′ E, 27°23′ N) by the 2050s, followed by a long-distance movement of 63.89 km southeast to Dutouqiao Town, Shuangqing District (111°35′ E, 27°14′ N), by the 2070s, and it subsequently displaces an additional 85.69 km northeast to Yutang Town, Xiangxiang City (112°20′ E, 27°37′ N), by the 2090s. These projected shifts reflect significant centroid repositioning in response to intensified climate change, highlighting the substantial spatial and temporal variability across different emission scenarios.

## 4. Discussion

### 4.1. Optimization of the MaxEnt Model and Validation of Prediction Accuracy

The predictive accuracy of species distribution models (SDMs) is highly dependent on the calibration of model parameters [66]. Unoptimized models are often associated with risks of overfitting or underfitting, resulting in either limited extrapolation capability due to excessive reliance on training data, or an oversimplified representation that fails to capture authentic ecological relationships [67]. In contrast to earlier studies that commonly employed default parameter settings, the current study systematically implemented parameter optimization using the ENMeval R package. This methodological rigor significantly improved model robustness, a benefit that has been consistently demonstrated in recent ecological studies applying similar parameter optimization frameworks [68,69]. The optimal parameter combination (RM = 4, FC = QHPT) yielded a Delta AICc value of 0, compared to 121.87 for the default parameter set. This substantial difference strongly supports the first hypothesis (H1) proposed in this study—that parameter optimization significantly improves model performance and controls overfitting. Similar approaches have been validated for other tree species, such as *Litsea cubeba* [49] and *Quercus gilva* [70], further affirming that parameter optimization is a critical step in enhancing the predictive capability of SDMs.

To comprehensively evaluate model accuracy, we adopted a multi-metric framework incorporating AUC, TSS, and Kappa coefficients to avoid potential biases associated with single-metric reliance [71]. An AUC value exceeding 0.9 indicated excellent discriminatory power, while complementary TSS and Kappa values further validated prediction consistency and accuracy, collectively affirming high model precision [52,53]. The classification threshold was determined using the Maximum Training Sensitivity Plus Specificity (MTSPS) method [54], which is widely recognized as a robust threshold-selection technique in species distribution modeling due to its insensitivity to species prevalence and effectiveness in balancing omission and commission errors [72]. Together, parameter optimization, multi-criteria validation, and threshold determination form a coherent and reproducible analytical framework. This methodology not only improves prediction reliability for *D. macropodum* but also provides a valuable reference for modeling distributions of phylogenetically or ecologically similar plant species.

### 4.2. Mechanisms of Dominant Environmental Variables in Driving the Suitable Habitat of D. macropodum

Understanding the environmental drivers of species distribution is considered a fundamental aspect of ecological niche modeling [73,74]. In this study, the current geographical distribution of *D. macropodum* was found to be jointly dominated by seasonal moisture and temperature factors. Among these, the precipitation of the coldest quarter (Bio19), diurnal temperature range (Bio2), and precipitation of the warmest quarter (Bio18) together accounted for the majority of the cumulative contribution, strongly supporting the second hypothesis (H2) of this study. As a key component of subtropical evergreen broad-leaved forests, the survival and reproduction of *D. macropodum* are highly dependent on stable hydrothermal conditions [18,20,21]. The high contribution of Bio19 indicates that water availability in winter is a critical limiting factor for the species. Winter drought can exacerbate physiological water stress, leading to membrane damage and carbon starvation, which is particularly critical for evergreen trees [75]. Meanwhile, Bio2, which reflects the magnitude of diurnal temperature variation, was identified as an indicator of the species’ adaptation to microclimatic stability. A lower diurnal temperature range is generally associated with frequent cloud cover and higher air humidity, which aligns with the observed preference of *D. macropodum* for moist valleys and shaded slopes in natural habitats, illustrating its physiological reliance on stable thermo-hydrological conditions [21].

Based on the analysis of univariate response curves generated by the MaxEnt model, we determined the required threshold ranges of key environmental variables to maintain a presence probability of at least 0.5 (for example, Bio2: 3.80–9.18 °C and Bio19: 484.65–2483.22 mm) [65]. These thresholds effectively quantify the core suitable ecological niche of the species under current climatic conditions and reflect key boundaries of its physiological tolerance. The high contribution of Bio19 underscores the critical importance of winter water availability for *D. macropodum*. Evergreens like *D. macropodum* face winter drought risk from transpiration amidst frozen soils, potentially causing xylem embolism [75]. Bio2 preference indicates adaptation to stable, humid microclimates that mitigate water stress, a hallmark of understory evergreens [21]. Under future climate change, continued deviation of temperature and precipitation from these optimal ranges is expected to lead to reduced fitness of *D. macropodum*, resulting in contraction and fragmentation of its suitable habitats. Notably, the contributions of topographic factors and the Human Footprint Index (HFI) were relatively low, a finding consistent with Sun et al. [76]. This does not imply that these factors are unimportant, but that they may be explained by two reasons: first, most occurrence records of *D. macropodum* are located in natural habitats with low human disturbance [18], where the HFI shows limited variation; second, the strong signal of climatic factors may have overshadowed the local effects of topography and human activities at macro-scales [46]. This further underscores the overwhelming role of climate change as a macro-scale driver.

### 4.3. Assessment of the Potential Distribution Dynamics of D. macropodum Under Climate Change Scenarios

Dynamic simulation of future species distribution patterns is recognized as a critical scientific foundation for assessing climate vulnerability and formulating targeted conservation strategies [77,78,79]. The projections from the multi-scenario ecological niche models in this study indicate that a significant reduction in suitable habitat area could be faced by *D. macropodum* between now and the end of this century. A consistent contraction trend in total suitable habitat was observed across all Shared Socioeconomic Pathways (SSPs), with the magnitude of loss being exacerbated by increased radiative forcing and prolonged time. Under the high-forcing SSP585 scenario, the highly suitable area is projected to decrease by over 80% by the 2090s, a loss significantly greater than that under the SSP126 pathway, which supports the third hypothesis (H3) of this study. This contraction trend is consistent with global patterns of range contraction driven by climate change [7]. As a specialist species with a narrow niche breadth, *D. macropodum* is characterized by low adaptability to environmental fluctuations, and is therefore considered more vulnerable to the adverse effects of climate change [80]. These projections highlight the necessity of identifying potential climate refugia beyond the species’ current range, exploring feasible routes for assisted migration, and developing targeted ecosystem adaptation and restoration strategies [81].

Spatial migration trajectories clearly reveal that current core suitable habitats are expected to undergo severe contraction, while new suitable areas are projected to expand toward higher elevations and latitudes, confirming the classic ecological response of climate tracking [8]. However, it has been further demonstrated that this migration is non-linear and fraught with uncertainties. Under high-emission scenarios, long-distance and non-linear shifts in the distribution centroid are observed, validating the fourth hypothesis (H4). This complex migration pattern may be attributed to more pronounced climate variability and increased frequency of extreme events under high-emission scenarios, resulting in increasingly fragmented and unpredictable spatial distributions of suitable habitats [82,83]. The ecological significance of these centroid shifts lies in their representation of the core trajectory of the species’ climatic niche movement. However, the centroid is a geometric abstraction, and its movement can be influenced by asymmetric habitat loss (e.g., contraction at the warm, trailing edge) and colonization (e.g., expansion at the cool, leading edge). Therefore, while the centroid provides a robust, integrated indicator of the overall range shift direction and magnitude, its interpretation must be coupled with observed patterns of habitat fragmentation and stability in order to fully assess the species’ vulnerability and dispersal challenges under climate change. Severe habitat fragmentation is expected to pose significant dispersal barriers, potentially confining populations to suboptimal habitats and elevating the risk of local extinction [84]. The extreme centroid shifts under SSP585 (e.g., 85.69 km) exceed the plausible dispersal capacity of the large-seeded, animal-dispersed *D. macropodum*, especially with intensified fragmentation. They signify severe climatic pressure and migration risk rather than achievable expansion, highlighting the critical need for assisted migration.

The primary practical value of this study is in its precise identification of key climate refugia, including the mountainous regions of central Taiwan and the Zhejiang–Fujian hills, using a three-dimensional “stable–loss–gain” analytical framework. These areas, often characterized by complex topography and relatively stable microclimates, can provide ongoing shelter for species under changing climates, and should be prioritized in biodiversity conservation planning [85]. Therefore, current conservation strategies should immediately focus on enhancing protection within these identified refugia and improving connectivity through the construction of ecological corridors [86,87,88]. Concurrently, experimental population reintroductions in expansion zones are recommended to inform future assisted-migration efforts [89]. This study not only offers spatially explicit conservation priorities and intervention pathways for *D. macropodum* but also provides a multi-scenario, multi-temporal assessment framework that can support adaptive management decisions for other subtropical evergreen broad-leaved forest species with similar ecological traits in China and globally. Implementation of these priorities faces socioeconomic barriers such as land use conflicts and coordination costs. Integrating findings with China’s Ecological Redline policy and corridor networks could enhance feasibility, secure policy support, and promote collaborative conservation under climate change.

### 4.4. Limitations of This Study and Future Research Directions

A systematic assessment of the distribution dynamics of *D. macropodum* under current and future climate scenarios was conducted in this study. However, five limitations must be clarified. First, the model was based on the static niche assumption, and neither eco-evolutionary adaptations nor biotic interactions were incorporated. This may have led to overestimation of climatic vulnerability and underestimation of long-term persistence potential. Second, while potential expansion zones were identified, the model did not incorporate key dispersal processes such as landscape connectivity, species-specific dispersal limitations, or propagule dispersal pathways. This omission introduces uncertainty in the prediction of realistic colonization potential under future climate conditions. Third, the reliance on a single climate model (BCC-CSM2-MR) may have introduced uncertainty in the future projections. Fourth, the centroid shift analysis overlooked habitat suitability variation by weighting all areas equally, potentially misrepresenting core ecological shifts by neglecting high-quality habitats. Finally, human activities were only represented by a static Human Footprint Index, without dynamically simulating the interactive effects between future land use/land cover changes and climate stressors. This limitation restricts the model’s explanatory power under compound pressure scenarios.

To address these limitations, future research should focus on the following directions: First, the development of integrated frameworks that combine population genetics data with dynamic range shift models to quantify the contributions of local adaptation and gene flow to niche evolution could enable a more objective assessment of adaptive resilience. Second, the integration of individual-based models (IBMs) or mechanistic dispersal models with circuit theory could explicitly simulate functional connectivity and delineate potential migration corridors in fragmented landscapes. This integration would substantially improve assessments of species reachability and population establishment within newly suitable habitats. Third, incorporation of multiple global climate models (GCMs) and downscaling techniques would better capture future climate variability, thereby improving the reliability of species distribution forecasts across different scenarios. Fourth, the adoption of suitability-weighted centroid analyses or utilization of spatial population models could more effectively capture the influence of habitat quality on range dynamics. Fifth, integration of high-resolution land use simulation data under Shared Socioeconomic Pathways (SSPs) could unravel multi-scale interactive mechanisms between climate change and human disturbances, and help to further develop early-warning and adaptive governance frameworks for ecosystems under multiple stressors. Advancements in these directions are expected to significantly enhance mechanistic understanding of species distribution responses and support the development of scientifically sound conservation strategies.

## 5. Conclusions

Employing a parameter-optimized MaxEnt model integrated with multi-source occurrence records and multidimensional environmental variables, this study systematically evaluated potential distribution shifts of *D. macropodum* under both current and future climate scenarios. The analysis revealed that the species’ distribution is predominantly governed by seasonal hydrothermal factors, with the diurnal temperature range (Bio2), precipitation of the coldest quarter (Bio19), and precipitation of the warmest quarter (Bio18) collectively contributing 88.0% of the total influence, thereby delineating the core ecological niche of this species. Projections under future climate conditions uniformly indicate habitat contraction, particularly under the high-emission SSP585 scenario, in which over 80% of highly suitable habitat is anticipated to be lost by the 2090s. The centroid of suitable habitats is expected to shift non-linearly toward higher latitudes and elevations, accompanied by pronounced habitat fragmentation, highlighting the complex and uncertain nature of the species’ response to climate change. Through spatial dynamic assessment, critical climate refugia were identified in the central mountains of Taiwan Province, the hilly regions of Zhejiang and Fujian, and the border area between western Hunan and eastern Guizhou. These regions should be prioritized for conservation, with ecological corridors established to improve habitat connectivity and reduce migration barriers. The findings not only clarify the mechanistic responses of *D. macropodum* to climatic changes but also offer scientifically grounded and spatially explicit guidance for the conservation of keystone species in subtropical evergreen broad-leaved forests in China, supporting regional biodiversity conservation and adaptive ecosystem management. This scalable, optimized MaxEnt framework enables predictive distribution modeling for other keystone or endangered species, particularly in biodiverse regions. It also supports cross-taxon vulnerability assessments and conservation strategy development under climate change, offering a transferable tool for global biodiversity protection and adaptive management.

## Figures and Tables

**Table 1:** Environmental variables used to predict the potential distribution of *Daphniphyllum macropodum*.

Category	Abbreviation	Environmental Variables	Units	VIF
Bioclimatic	Bio2	Mean diurnal range (Mean of monthly)	°C	6.58
	Bio3	Isothermality (Bio2/Bio7) (×100)		7.39
	Bio8	Mean temperature of wettest quarter	°C	5.84
	Bio15	Variation in precipitation seasonality		3.10
	Bio18	Precipitation of warmest quarter	mm	2.73
	Bio19	Precipitation of coldest quarter	mm	4.64
Topographic	Elevation	Elevation	m	9.45
	Aspect	Aspect	°	1.20
	Slope	Slope	°	1.92
Vegetation	NDVI	Normalized Difference Vegetation Index		2.45
Human	HFI	Human Footprint Index		1.86

**Table 2:** Variations in climatically suitable area for *Daphniphyllum macropodum* under different climate scenarios.

Period	Area (10^4^ km^2^)			Rate of Change (%)		
	Stability	Contraction	Expansion	Stability	Contraction	Expansion
2050s-SSP126	173.41	30.27	11.35	80.65	14.08	5.28
2050s-SSP245	176.83	26.86	15.58	80.64	12.25	7.11
2050s-SSP585	159.18	44.50	10.42	74.35	20.79	4.87
2070s-SSP126	182.01	21.71	16.09	82.80	9.88	7.32
2070s-SSP245	157.69	46.01	10.83	73.51	21.45	5.05
2070s-SSP585	142.34	61.32	6.92	67.59	29.12	3.29
2090s-SSP126	181.88	21.83	16.60	82.56	9.91	7.54
2090s-SSP245	144.23	59.41	10.00	67.51	27.81	4.68
2090s-SSP585	117.81	85.83	3.85	56.78	41.36	1.86

## Data Availability

The original contributions presented in this study are included in this article. Further inquiries can be directed to the corresponding author.

## Supplementary Materials

The following supporting information can be downloaded at: https://www.mdpi.com/article/10.3390/biology14101360/s1, Table S1: Twenty-four environmental variables used in this study; Figure S1: Spearman correlation matrix illustrating pairwise associations among the 24 environmental variables used in this study. The shape of each ellipse reflects the magnitude of the correlation, with progressively slender ellipses denoting higher absolute correlation coefficients. Color intensity encodes the direction of the relationship: deepening blue signifies increasingly negative correlations, while intensifying red indicates stronger positive associations.

## References

[1] Kim S.K., Shin J., An S.I., Kim H.J., Im N., Xie S.P., Kug J.S., Yeh S.W. (2022). Widespread irreversible changes in surface temperature and precipitation in response to CO_2_ forcing. Nat. Clim. Change.

[2] Shivanna K.R. (2022). Climate change and its impact on biodiversity and human welfare. Proc. Indian Natl. Sci. Acad..

[3] Trew B.T., Maclean I.M.D. (2021). Vulnerability of global biodiversity hotspots to climate change. Glob. Ecol. Biogeogr..

[4] Li H.L., Ali A., Luo X., Liao K., Sun N., Xu M.S., Sha L.B., He D., Du Y.J., Sun W.W. (2024). China’s subtropical deciduous plants are more sensitive to climate change than evergreen plants by flowering phenology. Glob. Change Biol..

[5] Zhou S., Yu B., Zhang Y. (2023). Global concurrent climate extremes exacerbated by anthropogenic climate change. Sci. Adv..

[6] Yin J., Gentine P., Slater L., Gu L., Pokhrel Y., Hanasaki N., Guo S., Xiong L., Schlenker W. (2023). Future socio-ecosystem productivity threatened by compound drought–heatwave events. Nat. Sustain..

[7] Pecl G.T., Araújo M.B., Bell J.D., Blanchard J., Bonebrake T.C., Chen I.C., Clark T.D., Colwell R.K., Danielsen F., Evengård B. (2017). Biodiversity redistribution under climate change: Impacts on ecosystems and human well-being. Science.

[8] Chen I.C., Hill J.K., Ohlemüller R., Roy D.B., Thomas C.D. (2011). Rapid Range Shifts of Species Associated with High Levels of Climate Warming. Science.

[9] Mi X., Feng G., Hu Y., Zhang J., Chen L., Corlett R.T., Hughes A.C., Pimm S., Schmid B., Shi S. (2021). The global significance of biodiversity science in China: An overview. Natl. Sci. Rev..

[10] Li D., Wu S., Liu L., Zhang Y., Li S. (2018). Vulnerability of the global terrestrial ecosystems to climate change. Glob. Change Biol..

[11] Lian X., Jiao L., Hu Y., Liu Z. (2023). Future climate imposes pressure on vulnerable ecological regions in China. Sci. Total Environ..

[12] Gao M., Hu Y., Bai Y. (2022). Construction of ecological security pattern in national land space from the perspective of the community of life in mountain, water, forest, field, lake and grass: A case study in Guangxi Hechi, China. Ecol. Indic..

[13] Liu D., Chang Q. (2015). Ecological security research progress in China. Acta Ecol. Sin..

[14] Zhang Z., Hu B., Jiang W., Qiu H. (2023). Construction of ecological security pattern based on ecological carrying capacity assessment 1990–2040: A case study of the Southwest Guangxi Karst-Beibu Gulf. Ecol. Model.

[15] Lyu L., Wang J., Wu H., Wang S., Zhou T., Xu Y., Wei X., Jiang M. (2022). Species composition and community structure of a mixed broadleaf-conifer forest dominated by *Liriodendron chinense* in the Jiugong Mountain, central China. Chin. J. Ecol..

[16] Wang L., Ouyang M., Song S., Zeng X., Song Q., Liu J., Fang X., Luan F., Yang Q. (2023). Response of N and P stoichiometric characteristics of evergreen broad-leaved forest plant leaf to *Phyllostachys edulis* expansion. Guihaia.

[17] Chen Z., Li Q., Jiang Z., Yan P., Arif M. (2024). Leaf functional traits of *Daphniphyllum macropodum* across different altitudes in Mao’er Mountain in Southern China. Front. For. Glob. Change.

[18] Tan Y., Zhang T., Jiang X., Shen W., Ye J. (2023). Altitudinal Variation Pattern in *Daphniphyllum macropodum* Leaf Traits and Influencing Environmental Factors in Mao’er Mountain, China. Chin. J. Appl. Ecol..

[19] Xu L.L., Niu Z.P., Chen D.Z., Zhang Y., Zhao Q., Liang H., Li S., Li J.L., Ding X., Yang C.L. (2024). Daphmacrimines A−K, Daphniphyllum alkaloids from *Daphniphyllum macropodum* Miq. Phytochemistry.

[20] Yao L., Ai X., Yi Y., Lu X. (2015). Study on the Population Characteristics of Ornamental Plant *Daphniphyllum macropodum* Miq. in Xingdoushan Nature Reserve. J. Hubei Univ. Natl. (Nat. Sci. Ed.).

[21] Zhao M., Pang C., Yang S., Luo Y., Yu S. (2015). Spatial patterns of *Daphniphyllum macropodum* in Tianmu Mountain, Zhejiang Province. J. Zhejiang Univ. (Sci. Ed.).

[22] Chen Z., Jiang Z., Li Q., Tan Y., Yan P., Arif M. (2024). Examining the stoichiometry of C:N:P:K in the dynamics of foliar-litter-soil within dominant tree species across different altitudes in southern China. Glob. Ecol. Conserv..

[23] Choi Y.-G., Park J.-H., Yoichi W., Oh S.-H. (2024). Hybridization and introgression in *Daphniphyllum macropodum* (Daphniphyllaceae) on Ulleungdo Island. J. Plant Biol..

[24] Abe H., Matsuki R., Ueno S., Nashimoto M., Hasegawa M. (2006). Dispersal of Camellia japonica seeds by Apodemus speciosus revealed by maternity analysis of plants and behavioral observation of animal vectors. Ecol. Res..

[25] Eljounaidi K., Radzikowska B.A., Whitehead C.B., Taylor D.J., Conde S., Davis W., Dowle A.A., Langer S., James S., Unsworth W.P. (2024). Variation of terpene alkaloids in *Daphniphyllum macropodum* across plants and tissues. New Phytol..

[26] Yoichi W., Matsuzawa S., Tamaki I., Nagano A.J., Oh S.-H. (2023). Genetic differentiation and evolution of broad-leaved evergreen shrub and tree varieties of *Daphniphyllum macropodum* (Daphniphyllaceae). Heredity.

[27] Phillips S.J., Anderson R.P., Schapire R.E. (2006). Maximum entropy modeling of species geographic distributions. Ecol. Model..

[28] Elith J., Phillips S.J., Hastie T., Dudík M., Chee Y.E., Yates C.J. (2011). A statistical explanation of MaxEnt for ecologists. Divers. Distrib..

[29] Elith J.H., Graham C.P., Anderson R., Dudík M., Ferrier S., Guisan A.J., Hijmans R., Huettmann F.R., Leathwick J., Lehmann A. (2006). Novel methods improve prediction of species’ distributions from occurrence data. Ecography.

[30] Anderson R.P., Gonzalez I. (2011). Species-specific tuning increases robustness to sampling bias in models of species distributions: An implementation with Maxent. Ecol. Model..

[31] Kramer-Schadt S., Niedballa J., Pilgrim J.D., Schröder B., Lindenborn J., Reinfelder V., Stillfried M., Heckmann I., Scharf A.K., Augeri D.M. (2013). The importance of correcting for sampling bias in MaxEnt species distribution models. Divers. Distrib..

[32] Fang H.Q., Zhang P.F., Xu S.W., Xu T., He B., Wang E., Dong C.W., Yang Q.S. (2024). The ecological suitability area of *Cirsium lineare* (Thunb.) Sch.-Bip. under future climate change in China based on MaxEnt modeling. Ecol. Evol..

[33] Fang B., Zhao Q., Qin Q., Yu J. (2022). Prediction of Potentially Suitable Distribution Areas for *Prunus tomentosa* in China Based on an Optimized MaxEnt Model. Forests.

[34] Dong Z., Jiang H., Zhang W., Wu J., Yang Y., Yang T., Zhao J., Luo C., Yang X., Li G. (2025). Potential distribution prediction of *Terminalia chebula* Retz. in China under current and future climate scenarios. Ecol. Evol..

[35] Di Musciano M., Di Cecco V., Bartolucci F., Conti F., Frattaroli A.R., Di Martino L. (2020). Dispersal ability of threatened species affects future distributions. Plant Ecol..

[36] Weeks T.L., Betts M.G., Pfeifer M., Wolf C., Banks-Leite C., Barbaro L., Barlow J., Cerezo A., Kennedy C.M., Kormann U.G. (2023). Climate-driven variation in dispersal ability predicts responses to forest fragmentation in birds. Nat. Ecol. Evol..

[37] Muscarella R., Galante P.J., Soley-Guardia M., Boria R.A., Kass J.M., Uriarte M., Anderson R.P. (2014). ENMeval: An R package for conducting spatially independent evaluations and estimating optimal model complexity for Maxent ecological niche models. Methods Ecol. Evol..

[38] Zhang L., Yang C., Wang P., Xie G., Wang W. (2025). Climate change and geographic barriers exacerbate the spread and threat of *Psacothea hilaris* (Pascoe, 1857) in China: Insights from ensemble model, geographic barrier simulations, and niche analysis. Sci. Total Environ..

[39] Global Biodiversity Information Facility (GBIF).

[40] Aiello-Lammens M.E., Boria R.A., Radosavljevic A., Vilela B., Anderson R.P. (2015). spThin: An R package for spatial thinning of species occurrence records for use in ecological niche models. Ecography.

[41] Varela S., Anderson R.P., García-Valdés R., Fernández-González F. (2014). Environmental filters reduce the effects of sampling bias and improve predictions of ecological niche models. Ecography.

[42] Didan K. (2015). MODIS/Terra Vegetation Indices Monthly L3 Global 1km SIN Grid V006.

[43] Mu H., Li X., Wen Y., Huang J., Du P., Su W., Miao S., Geng M. (2022). A global record of annual terrestrial Human Footprint dataset from 2000 to 2018. Sci. Data.

[44] Jin H., Qiao L., Wang S., Kong L., Zhang J. (2024). Performance evaluation of surface air temperature simulated by the Beijing Climate Central Climate Model based on the climate complexity. Clim. Dyn..

[45] Liao D., Zhou B., Xiao H., Zhang Y., Zhang S., Su Q., Yan X. (2025). MaxEnt Modeling of the Impacts of Human Activities and Climate Change on the Potential Distribution of *Plantago* in China. Biology.

[46] Dormann C.F., Elith J., Bacher S., Buchmann C., Carl G., Carré G., Marquéz J.R.G., Gruber B., Lafourcade B., Leitão P.J. (2013). Collinearity: A review of methods to deal with it and a simulation study evaluating their performance. Ecography.

[47] Kass J.M., Muscarella R., Galante P.J., Bohl C.L., Pinilla-Buitrago G.E., Boria R.A., Soley-Guardia M., Anderson R.P. (2021). ENMeval 2.0: Redesigned for customizable and reproducible modeling of species’ niches and distributions. Methods Ecol. Evol..

[48] Phillips S.J., Dudík M. (2008). Modeling of species distributions with Maxent: New extensions and a comprehensive evaluation. Ecography.

[49] Shi X., Wang J., Zhang L., Chen S., Zhao A., Ning X., Fan G., Wu N., Zhang L., Wang Z. (2023). Prediction of the potentially suitable areas of *Litsea cubeba* in China based on future climate change using the optimized MaxEnt model. Ecol. Indic..

[50] Xu C., Zhang L., Zhang K., Tao J. (2023). MaxEnt Modeling and the Impact of Climate Change on *Pistacia chinensis* Bunge Habitat Suitability Variations in China. Forests.

[51] Li Y., Li M., Li C., Liu Z. (2020). Optimized Maxent model predictions of climate change impacts on the suitable distribution of *Cunninghamia lanceolata* in China. Forests.

[52] Allouche O., Tsoar A., Kadmon R. (2006). Assessing the accuracy of species distribution models: Prevalence, kappa and the true skill statistic (TSS). J. Appl. Ecol..

[53] Zhuo Z., Xu D., Pu B., Wang R., Ye M. (2020). Predicting distribution of *Zanthoxylum bungeanum* Maxim. in China. BMC Ecol..

[54] Yang L., Zhu X., Song W., Shi X., Huang X. (2024). Predicting the potential distribution of 12 threatened medicinal plants on the Qinghai-Tibet Plateau, with a maximum entropy model. Ecol. Evol..

[55] da Silva N.R., Souza P.G., de Oliveira G.S., da Silva Santana A., Bacci L., Silva G.A., Barry E.J., de Aguiar Coelho F., Soares M.A., Picanço M.C. (2024). A MaxEnt Model of Citrus Black Fly *Aleurocanthus woglumi* Ashby (*Hemiptera: Aleyrodidae)* under Different Climate Change Scenarios. Plants.

[56] Wen Z., Yan K., Zhang M., Ma R., Zhu X., Duan Q., Jiang X. (2024). Predicting the potential distribution of *Astragali radix* in China under climate change adopting the MaxEnt model. Front. Plant Sci..

[57] Xie Y., Huang H., Chen L., Xiao J., Weng F., Liu J., He T., Chen L., Rong J., Chen L. (2024). Forecasting appropriate habitats for rare and endangered *indocalamus Species* in China in response to climate change. Forests.

[58] Xiang Y., Yang Q., Li S., Liu Y., Li Y., Ren J., Yao J., Luo X., Luo Y., Yao B. (2025). Climate Change Drives Northwestward Migration of *Betula alnoides*: A Multi-Scenario MaxEnt Modeling Approach. Plants.

[59] Liu C., White M., Newell G. (2013). Selecting thresholds for the prediction of species occurrence with presence-only data. J. Biogeogr..

[60] Hou J., Xiang J., Li D., Liu X. (2023). Prediction of Potential Suitable Distribution Areas of *Quasipaa spinosa* in China Based on MaxEnt Optimization Model. Biology.

[61] Xu Y., Su X., Ren Z. (2024). Prediction of historical, current and future potential distribution of *Rhus chinensis* (Anacardiaceae) based on the optimized MaxEnt model in China. Plant Ecol..

[62] Tu G.H., Guo X.D., Xi S.Y., Ma X.H., Jin L. (2025). Predicting potential suitable habitat of *Cistanche deserticola* by integrating parasitic constraints and land use data into MaxEnt modeling. Front. Plant Sci..

[63] Brown J.L., Bennett J.R., French C.M. (2017). SDMtoolbox 2.0: The next generation Python-based GIS toolkit for landscape genetic, biogeographic and species distribution model analyses. PeerJ.

[64] Lu Z., Wang G., Shao Y., Yan L., Huang L., Fan Z., Han S., Ren X., Han R., Zhang C. (2025). Assessing the impacts of climate change and human activities on distribution of *Lophatherum gracile* in China using the maxent model. Sci. Rep..

[65] Liu Q., Liu L., Xue J., Shi P., Liang S. (2025). Habitat Suitability Shifts of *Eucommia ulmoides* in Southwest China Under Climate Change Projections. Biology.

[66] Piirainen S., Lehikoinen A., Husby M., Kålås J.A., Lindström Å., Ovaskainen O. (2023). Species distributions models may predict accurately future distributions but poorly how distributions change: A critical perspective on model validation. Divers. Distrib..

[67] Warren D.L., Seifert S.N. (2011). Ecological niche modeling in Maxent: The importance of model complexity and the performance of model selection criteria. Ecol. Appl..

[68] Rehan M., Hassan A., Zeb S., Ullah S., Ahmad F., Bohnett E., Bosso L., Fida T., Kabir M. (2024). Application of species distribution models to estimate and manage the Asiatic black bear (*Ursus thibetanus*) habitat in the Hindu Kush Mountains, Pakistan. Eur. J. Wildl. Res..

[69] Zhang J., Li X., Li S., Yang Q., Li Y., Xiang Y., Yao B. (2025). MaxEnt Modeling of Future Habitat Shifts of *Itea yunnanensis* in China Under Climate Change Scenarios. Biology.

[70] Shi J., Xia M., He G., Gonzalez N.C.T., Zhou S., Lan K., Ouyang L., Shen X., Jiang X., Cao F. (2024). Predicting *Quercus gilva* distribution dynamics and its response to climate change induced by GHGs emission Through MaxEnt modeling. J. Environ. Manag..

[71] Leroy B., Delsol R., Hugueny B., Meynard C.N., Barhoumi C., Barbet-Massin M., Bellard C. (2018). Without quality presence–absence data, discrimination metrics such as TSS can be misleading measures of model performance. J. Biogeogr..

[72] Freeman E.A., Moisen G.G. (2008). A comparison of the performance of threshold criteria for binary classification in terms of predicted prevalence and kappa. Ecol. Model..

[73] Ren Z., Zhao W., Chen N., Zhou X. (2024). Explaining the mechanisms behind niche dimensionality and light-driving species diversity based on functional traits. npj Biodiversity.

[74] Richards D., Lavorel S. (2023). Niche theory improves understanding of associations Between ecosystem services. One Earth.

[75] Adams H.D., Zeppel M.J.B., Anderegg W.R.L., Hartmann H., Landhäusser S.M., Tissue D.T., Huxman T.E., Hudson P.J., Franz T.E., Allen C.D. (2017). A multi-species synthesis of physiological mechanisms in drought-induced tree mortality. Nat. Ecol. Evol..

[76] Sun J., Feng L., Wang T., Tian X., He X., Xia H., Wang W. (2021). Predicting the Potential Habitat of Three Endangered Species of *Carpinus* Genus under Climate Change and Human Activity. Forests.

[77] Trisos C.H., Merow C., Pigot A.L. (2020). The projected timing of abrupt ecological disruption from climate change. Nature.

[78] Jones K.R., Watson J.E.M., Possingham H.P., Klein C.J. (2016). Incorporating climate change into spatial conservation prioritisation: A review. Biol. Conserv..

[79] Duncanson L., Liang M., Leitold V., Armston J., Krishna Moorthy S.M., Dubayah R., Costedoat S., Enquist B.J., Fatoyinbo L., Goetz S.J. (2023). The effectiveness of global protected areas for climate change mitigation. Nat. Commun..

[80] Urban M.C. (2015). Accelerating extinction risk from climate change. Science.

[81] Hannah L., Roehrdanz P.R., Marquet P.A., Enquist B.J., Midgley G., Foden W., Lovett J.C., Corlett R.T., Corcoran D., Butchart S.H.M. (2020). 30% land conservation and climate action reduces tropical extinction risk by more than 50%. Ecography.

[82] Wu S., Luo M., Lau G.N.-C., Zhang W., Wang L., Liu Z., Lin L., Wang Y., Ge E., Li J. (2025). Rapid flips between warm and cold extremes in a warming world. Nat. Commun..

[83] Nie Y., Sun Y., Zhang X., Chen G. (2025). Human-induced changes in extreme cold surges across the Northern Hemisphere. Nat. Commun..

[84] Travis J.M.J., Delgado M., Bocedi G., Baguette M., Bartoń K., Bonte D., Boulangeat I., Hodgson J.A., Kubisch A., Penteriani V. (2013). Dispersal and species’ responses to climate change. Oikos.

[85] Keppel G., Van Niel K.P., Wardell-Johnson G.W., Yates C.J., Byrne M., Mucina L., Schut A.G.T., Hopper S.D., Franklin S.E. (2012). Refugia: Identifying and understanding safe havens for biodiversity under climate change. Glob. Ecol. Biogeogr..

[86] Cazzolla Gatti R. (2025). Ecological Peace Corridors: A new conservation strategy to protect human and biological diversity. Biol. Conserv..

[87] Beger M., Metaxas A., Balbar A.C., McGowan J.A., Daigle R., Kuempel C.D., Treml E.A., Possingham H.P. (2022). Demystifying ecological connectivity for actionable spatial conservation planning. Trends Ecol. Evol..

[88] Fida T., Mohammadi A., Almasieh K., Bosso L., Ud Din S., Shamas U., Nawaz M.A., Kabir M. (2025). Species distribution modelling and landscape connectivity as tools to inform management and conservation for the critically endangered Himalayan brown bear (*Ursus arctos isabellinus*) in the Deosai National Park, Pakistan. Front. Ecol. Evol..

[89] Hoegh-Guldberg O., Hughes L., McIntyre S., Lindenmayer D.B., Parmesan C., Possingham H.P., Thomas C.D. (2008). Assisted Colonization and Rapid Climate Change. Science.

