# Supplementary material for: Modeling the Habitat Suitability and Range Shift of Daphniphyllum macropodum in China Under Climate Change Using an Optimized MaxEnt Model
Source: Biology (Basel). 2025 Oct 3;14(10):1360. doi: 10.3390/biology14101360 (PMC12561130; doi:10.3390/biology14101360)
Supplement: Supplementary file 1 [file biology-14-01360-s001.zip › biology-3882754-supplementary.pdf]

Supplementary Materials

Modeling the Habitat Suitability and Range Shift of *Daphniphyllum macropodum* in China Under Climate Change Using an Optimized MaxEnt Model

Yangzhou Xiang [1], Suhang Li [1], Qiong Yang [1], Jiaojiao Liu [1], Ying Liu [2], Ling Zhao [3], Hua Lin [1], Yang Luo [1], Jun Ren [1], Xuqiang Luo [1,*] and Hua Wang [4,*]

[1] School of Geography and Resources, Guizhou Education University, Guiyang 550018, China
[2] School of Biological Sciences, Guizhou Education University, Guiyang 550018, China
[3] State Power Investment Corporation Power Station Operation Technology (Beijing) Co., Ltd., Beijing 100032, China
[4] Guizhou Institute of Forest Inventory and Planning, Guiyang 550003, China
* Correspondence: xuqiangluo@gznc.edu.cn (X.L.); hualywang@126.com (H.W.)

Table S1. Twenty-four environmental variables used in this study.

Category	Abbreviation	Environmental variables	Units	Range
Bioclimatic	Bio1	Annual mean temperature	°C	9.31～22.39
	Bio2	Mean diurnal range (Mean of monthly)	°C	5.09～12.56
	Bio3	Isothermality (Bio2/Bio7) (× 100)		23.27～48.28
	Bio4	Standard deviation of temperature seasonality		304.86～908.53
	Bio5	Max temperature of warmest month	°C	16.42～34.29
	Bio6	Min temperature of coldest month	°C	-4.47～11.19
	Bio7	Temperature annual range (Bio5-Bio6)	°C	14.03～33.84
	Bio8	Mean temperature of wettest quarter	°C	12.65～28.08
	Bio9	Mean temperature of driest quarter	°C	0.97～18.30
	Bio10	Mean temperature of warmest quarter	°C	13.38～28.48
	Bio11	Mean temperature of coldest quarter	°C	0.61～15.25
	Bio12	Annual precipitation	mm	720.00～3613.00
	Bio13	Precipitation of wettest month	mm	154.00～738.00
	Bio14	Precipitation of driest month	mm	6.00～183.00
	Bio15	Variation of precipitation seasonality		24.11～98.19
	Bio16	Precipitation of wettest quarter	mm	417.00～1735.00
	Bio17	Precipitation of driest quarter	mm	22.00～582.00
	Bio18	Precipitation of warmest quarter	mm	332.00～1712.00
	Bio19	Precipitation of coldest quarter	mm	22.00～601.00
Topographic	Elevation	Elevation	m	11.00～2561.00
	Aspect	Aspect	°	1.30～359.27
	Slope	Slope	°	0.01～8.25
Vegetation	NDVI	Normalized difference vegetation index		0.04～0.88
Human	HFI	Human footprint index		1.58～49.92

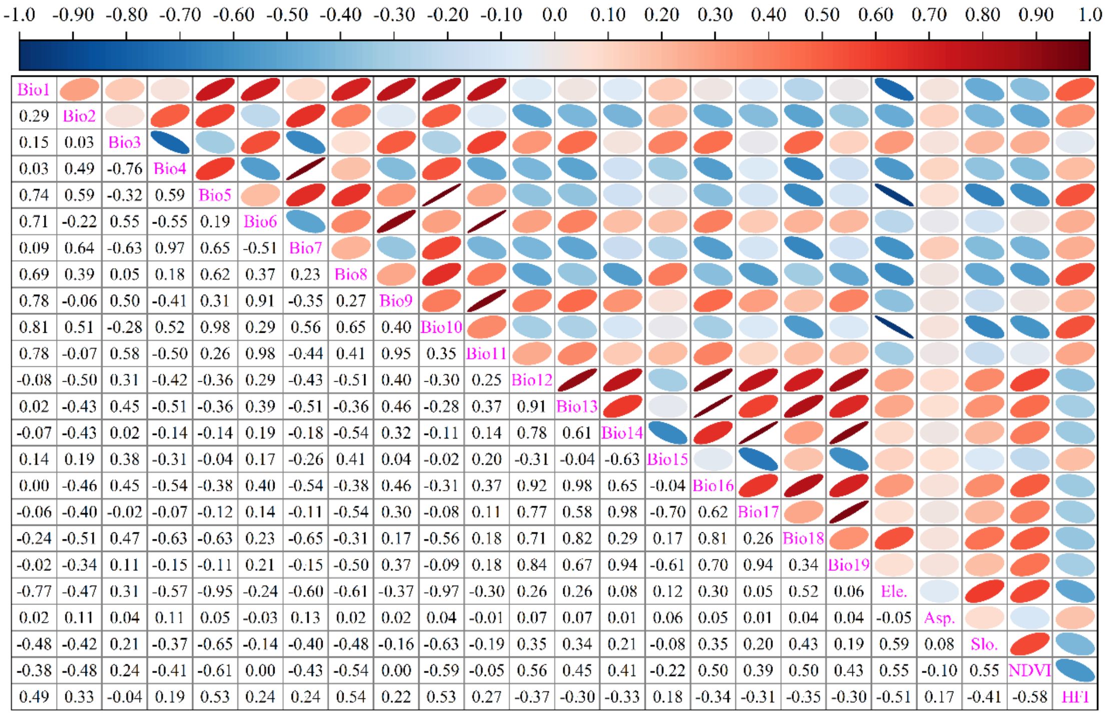

Figure S1. Spearman correlation matrix illustrating pairwise associations among the 24 environmental variables used in this study. The shape of each ellipse reflects the magnitude of the correlation, with progressively slender ellipses denoting higher absolute correlation coefficients. Color intensity encodes the direction of the relationship: deepening blue signifies increasingly negative correlations, while intensifying red indicates stronger positive associations. The abbreviations Ele., Asp., and Slo. correspond to Elevation, Aspect, and Slope, respectively.
